# Morphological and Molecular Characterization of Two Species of *Ligophorus* (Monogenea: Ancyrocephalidae) in Mullets from the Yucatán Peninsula, with Comments on the Geographical Distribution of *L*. *mediterraneus*

**DOI:** 10.1007/s11686-024-00953-5

**Published:** 2025-01-24

**Authors:** Leopoldo Andrade-Gómez, Reinaldo José da Silva, Gerardo Pérez-Ponce de León

**Affiliations:** 1 Departamento de Sistemas y Procesos Naturales, Escuela Nacional de Estudios Superiores Unidad Mérida, Km 4.5 Carretera Mérida-Tetiz, Ucú, Yucatán C.P. 97357 Mexico; 2 Institute of Biosciences, Section Parasitology, São Paulo State University (UNESP), Botucatu, São Paulo 18618-689 Brazil

**Keywords:** Morphology, DNA, Ribosomal genes, Gulf of Mexico, *Mugil*

## Abstract

**Background:**

*Ligophorus* Euzet and Suriano, 1977 is a specious genus of ancyrocephalid monogeneans parasitizing mullets around the world, with most species distributed in the western Pacific and the Mediterranean Sea. Only nine out of the 62 species in the genus have been reported from the Americas, and from them, only two have been sequenced.

**Methods:**

We analyzed two species of *Mugil* (L.) from Northern Yucatán Peninsula. Specimens of *Ligophorus* were sampled from the gills of their hosts. The morphology of the specimens was examined. In addition, 28S and ITS rDNA sequences were obtained and compared with previous sequences downloaded from GenBank.

**Results:**

We discovered two species of *Ligophorus* using morphological and molecular characters, *L. mediterraneus*, parasitizing the stripped mullet *Mugil cephalus* off the coast of Celestún, and *L. yucatanensis*, parasitizing the silver mullet *M. curema* in four coastal lagoons. Sequence data of the latter species are reported for the first time.

**Conclusion:**

Our findings showed that two species of *Ligophorus* occur in mugilids of the Yucatán Peninsula. One represents a widely distributed marine species with records in the Mediterranean Sea and the Yucatán Peninsula, whereas the second one, *L. yucatanensis*, represents an endemic species restricted to coastal lagoons of the Yucatán Peninsula.

**Supplementary Information:**

The online version contains supplementary material available at 10.1007/s11686-024-00953-5.

## Introduction

*Ligophorus* Euzet and Suriano 1977 is one of the most species-rich genera among the Ancyrocephalidae Bychowsky, 1937, with 62 nominal species distributed worldwide [1, 2]. Species of *Ligophorus* are exclusively parasites of mullets (Mugilidae) and most of them have been described from members of the genera *Planiliza* Whitley and *Mugil* (L.), with 23 and 19 species reported thus far, respectively. Most species (80%) have been described from Asia and the Mediterranean Sea, including *Ligophorus mediterraneus* Sarabeev, Balbuena & Euzet, 2005 ex *Mugil cephalus* (L.) [1]. In the Americas, only nine species of *Ligophorus* have been reported from mugilids inhabiting marine, estuarine or freshwater habitats. Seven of these species were described in South America. In contrast, only two species have been reported in North America as parasites of *M. cephalus* in the Gulf of Mexico, i.e., *L. mugilinus* (Hargis, 1955), and *L. yucatanensis* Rodríguez-González, Míguez-Lozano, Llopis-Belenguer & Balbuena, 2015 [3].

The genetic library for species in the genus has increased significantly in the last few years. Thirty-one species of *Ligophorus* have been sequenced for the 28S rRNA gene, 21 species for ITS1, and 14 species for 18S rDNA [4–7]; phylogenetic analyses for species in the genus were recently published and showed a lack of association between the species of *Ligophorus* and the host species and geographical distribution [8, 9]. In addition, the three molecular markers resulted in different topologies most likely due to a sample size artifact [9].

Still, there is a paucity of molecular data for species reported from the Americas since only two of the nine species are sequenced, *L. saladensis* Marcotegui & Martorelli, 2009 and *L. uruguayensis* Siquier & Ostrowski de Núñez, 2009 [5]. In this study, we aimed to characterize morphologically and molecularly the species of *Ligophorus* sampled from the gills of the grey mullet, *M. cephalus*, and the silver mullet, *M. curema* from offshore and coastal lagoons of Yucatán, respectively.

## Materials and Methods

### Host Collection

**Fig. 1:**
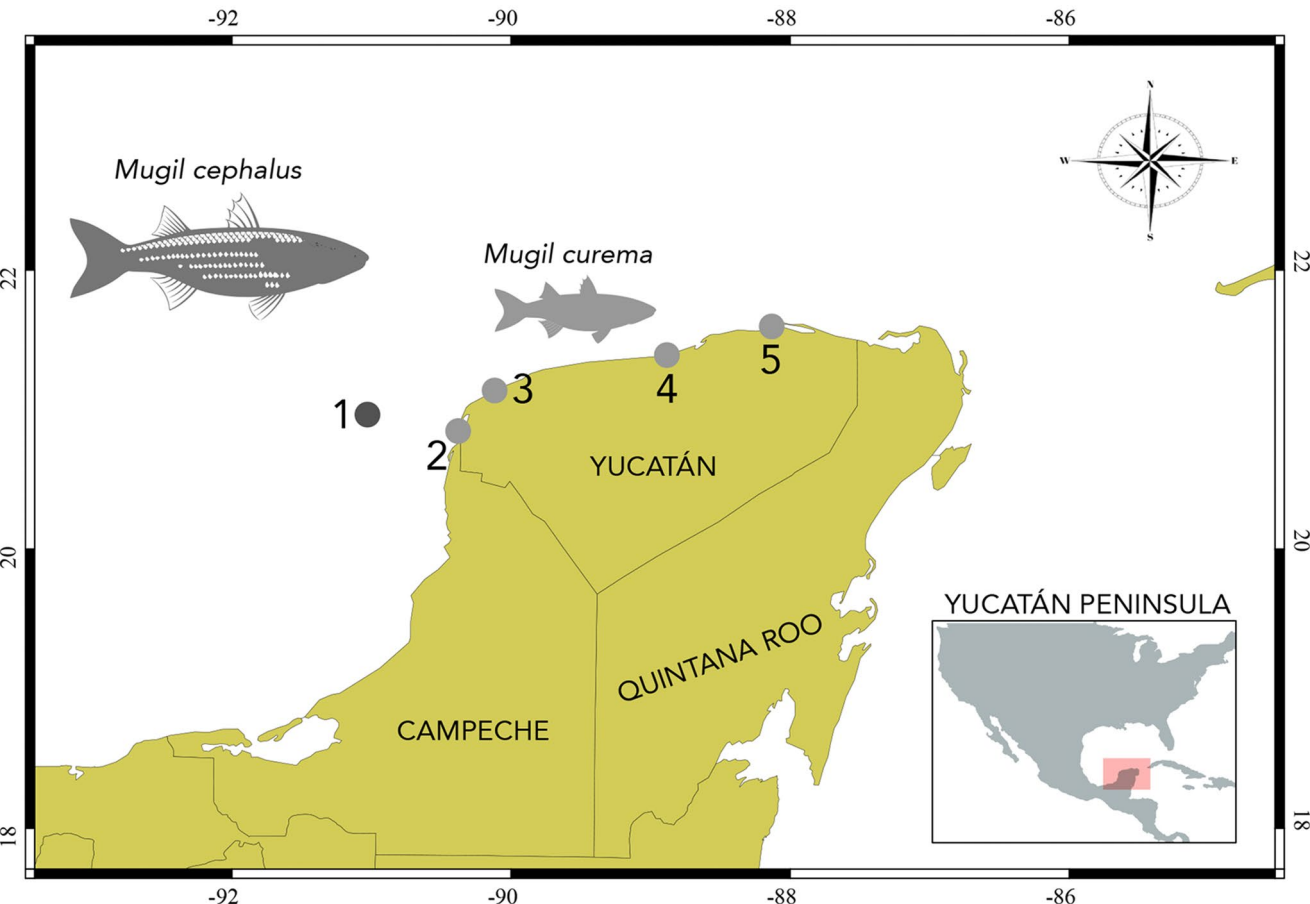
Samplig collection in the Yucatán Peninsula, Mexico. (1) Adult specimens of *Mugil cephalus* collected off Celestún, Yucatán. (2–5) Juvenile specimens of *Mugil curema* collected in coastal lagoons in Yucatán. Localities correspond with Table 1

Four adult specimens of *M. cephalus* were obtained from the commercial capture in April 2023 offshore of Celestún, Yucatán State, Mexico, and kept on ice. In addition, 57 juvenile specimens of *M. curema* were collected from four coastal lagoons of Yucatán using cast nets, and were kept alive until necropsied (Table 1; Fig. 1). Individual silver mullets were euthanized by spinal severance (pithing) following the procedures accepted by the American Veterinary Medical Association [10], dissected, and immediately examined under a stereomicroscope. Specimens of *Ligophorus* were recovered from the gills of both species of mullets (Table 1). Specimens were fixed in hot distilled water and preserved in 100% ethanol for morphological and molecular analyses.

**Table 1:** *Ligophorus* spp. recorded in this study with locality, host species, host length, prevalence and range of intensity according to Bush et al. [33] and Genbank accession number. TL = total length of hosts; P = prevalence, HI/HR = host infected/Host revised; RI = Range of intensity. Numbers for localities (NL), which correspond to Fig. 1

NL	Locality	Georeference	Host	TL (cm)	*P*% (HI/HR)	RI	Species of Ligophorus	28S	ITS1
1	Off Celestún	20° 58′ 9.6′′ N, 91° 3′ 9.1′′ W	*Mugil cephalus*	48–52	100 (4/4)	10–18	* L. mediterraneus*	PQ634769–773	PQ634786–788
2	Celestún	20° 50′ 53.5′′ N, 90° 24′ 22′′ W	*Mugil curema*	10–18	40 (4/10)	3–8	* L. yucatanensis*	PQ634774	PQ634789
3	La Carbonera	21° 08′ 1.5′′ N, 90° 07′ 55.9′′ W	*Mugil curema*	8–12	25 (2/8)	5–8	* L. yucatanensis*	PQ634775–778	PQ634790
4	Dzilam de Bravo	21° 23′ 39.7′′ N, 88° 53′ 20.6′′ W	*Mugil curema*	16–21	38 (8/21)	9–26	* L. yucatanensis*	PQ634779–781	–
5	Ría Lagartos	21° 35′ 47.3′′ N, 88° 8′ 44.6′′ W	*Mugil curema*	9–12	89 (16/18)	9–25	* L. yucatanensis*	PQ634782–785	PQ634791

## Morphological Analysis

Twenty specimens were mounted in Hoyer or Gray & Wess medium to study the sclerotized structures [11]. Morphometrical analyses were made using a computerized microscope equipped with a system for image analysis with differential interference contrast (DIC) and phase contrast LAS V3 (Leica Application Suite V3; Leica Microsystems, Wetzlar, Germany). Measurements are given in micrometres (µm). Voucher specimens were deposited under the number 764–782 L at the Coleção Helmintológica do Instituto de Biociencias (CHIBB), Universidade Estadual Paulista (UNESP), Botucatu, São Paulo State, Brazil; and at the Colección Nacional de Helmintos (CNHE), UNAM, Mexico City under the number 12,161–12,162.

## Molecular Analysis

Seventeen specimens of *Ligophorus* spp. collected from *M. curema* and *M. cephalus* were placed individually overnight in tubes with a digestion solution for DNA extraction at 56°C. The digestion procedure, amplification, and sequencing protocols followed Andrade-Gómez et al. [12]. The domains D1–D3 of the large subunit of nuclear ribosomal RNA gene (28S) and sequences of the Internal Transcribed Spacers rDNA (ITS1) were amplified via PCR using the primers: 391 5’– AGCGGAGGAAAAGAAACTAA–3’, plus 536: 5’–CAGCTATCCTGAGG GAAAC–3’ for 28S [13]; and BD1 5’–GTCGTAACAAGGTTTCCGTA– 3’, plus BD2 5’–TATGCTTAAATTCAGCGGGT– 3’ for ITS [14]. Sequencing internal primers were 502 plus 503 for 28S [13, 15]; and BD3 plus BD4 for ITS1 [16]. Sequences were assembled and edited using Geneious v7 [17] and deposited in the GenBank database.

The newly obtained sequences were aligned independently with those from other *Ligophorus* spp. downloaded from GenBank, plus *Ergenstrema mugilis* Paperna, 1964 used as outgroup for rooting the trees (see Table 2). Alignments were built using the software Clustal W [18] with default parameters and adjusted manually with the Mesquite software [19]. The alignment of the 28S dataset consisted of 67 sequences with 1,007 nucleotides. The ITS1 alignment consisted of 52 sequences with 892 nucleotides.

**Table 2:** Sequences of *Ligophorus* spp. from GenBank used for phylogenetic analyses in the present study. New sequences generated in the present study are in bold

Host species	Ligophorus spp.	Locality	28S	ITS	Reference
*Chelon auratus*	*L. szidati*	Mediterranean Sea, Ebro Delta	JN996806	JN996841	[4]
	*L. vanbenedenii*		JN996801–02	JN996836–37	
*Chelon labrosus*	*L. angustus*	Mediterranean Sea, off Cullera	JN996803, 05	JN996838–40	
*Chelon ramado*	*L. confusus*	Mediterranean Sea, off Cullera, Ebro Delta	JN996807–08, 10	JN996842, 47	
	*L. imitans*		JN996814	JN996849–51	
*Chelon saliens*	*L. acuminatus*	Mediterranean Sea, Ebro Delta	JN996816	JN996852	
	*L. heteronchus*		JN996812	JN996848	
	*L. macrocolpos*		JN996819–21	JN996855–56	
	*L. minimus*		JN996817–18	JN996853–54	
*Crenimugil buchanani*	*L. fenestrum*	Indian Ocean, Strait of Malacca, Langkawi Island	KM221913	KM221926	[6]
	*L. kedahensis*		KM221917	KM221929	
	*L. kederai*		KM221918	–	
	*L. grandis*	Indian Ocean, Strait of Johor, Malaysia	KM221915	–	
	*L. johorensis*		KM221916	–	
	*L. liewi*		KM221919	KM221931	
	*L. satunensis*	Satun, Thailand	–	MG922107	[34]
*Mugil cephalus*	*L. cephali*	Mediterranean Sea, off Cullera, Albufera	JN996830	JN996865; KP294376, 83	[4, 35]
	*L. chabaudi*	Mediterranean Sea, Ebro Delta	JN996831–34	JN996866–69	
	*L. mediterraneus*	Mediterranean Sea, off Cullera	JN996827–29	JN996862–64	
		Offshore of Celestún, Yucatán, Mexico	**PQ634769–773**	**PQ634786–788**	Present study
	*L. leporinus*	South China Sea, off Guangdong, China	DQ537380	–	[35]
*Mugil curema*	*L. yucatanensis*	Off Celestún, Yucatán, Mexico	**PQ634774**	**PQ634789**	Present study
		Off Carbonera, Yucatán, Mexico	**PQ634775–778**	**PQ634790**	
		Off Dzilam, Yucatán, Mexico	**PQ634779–781**	–	
		Off Ria Lagartos, Yucatán, Mexico	**PQ634782–785**	**PQ634791**	
*Mugil liza*	*L. saladensis*	Atlantic Ocean, off Brazil	KF442628–29	KF442627	[5]
	*L. uruguayensis*		KF442630	KF442626	
*Planiliza haematocheilus*	*L. kaohsianghsieni*	Black Sea, off Karadag	KY979156	MZ648433	[8]
		Sea of Japan Tavrichan Bay, mouth of River Kievka	MZ648426	MZ648430	
		Sea of Japan, Tavrichan Bay, mouth of River Razdolnaya	–	MZ648429	
	*L. llewellyni*	Sea of Azov, Utlyuksky Estuary	JN996822–23	JN996858	[4]
	*L. pilengas*		JN996824–26	JN996859–61	
		Black Sea, off Karadag	KY979153	–	[8]
*Planiliza subviridis*	*L. bantingensis*	Indian Ocean, Straits of Malacca, Carey Island, Selangor	KM221909	KM221922	[6]
	*L. belanaki*		KM221910	KM221923	
	*L. careyensis*		KM221911	KM221924	
	*L. chelatus*		KM221912	KM221925	
	*L. funnelus*		KM221914	–	
	*L. navjotsodhii*		KM221920	KM221932	
	*L. parvicopulatrix*		KM221921	–	

**Table 3:** Comparative metrical data for *L. Mugilinus* and *L. Mediterraneus*

Species		*L. mugilinus*	*L. mugilinus*	*L. mugilinus*	*L. mediterraneus*	*L. mediterraneus (Syn. L. mugilinus)*	*L. mediterraneus*	*L. mediterraneus*	*L. mediterraneus*
Reference		[30, 36]	[36]	[28]	Present study	[30]	[37]	[37]	[27]
Locality		Alligator Harbor, Florida, USA	Northwest Atlantic, Charleston, USA	Northwest Atlantic, Charleston, USA	Offshore Celestún, Atlantic Sea	Mediterranean Sea	Mediterranean Sea	Mediterranean Sea and Black Sea	Mediterranean Sea and Black Sea
Host		*Mugil cephalus*	*Mugil cephalus*	*Mugil cephalus*	*Mugil cephalus*	*Mugil cephalus*	*Mugil cephalus*	*Mugil cephalus*	*Mugil cephalus*
n	**	5	9	5	8	20	5	23	31
VENTRAL ANCHOR									
inner length (VI)	VAA	32 − 36	30 − 38	36 − 39	29 − 39 (35)	32 − 34	34.2	32 − 39	32 − 39
main part length (VM)	VAB	22 − 25	24 − 27	24 − 27	23 − 31 (26)	23 − 25	24.2	24 − 28	24 − 28
distal part length (VD)	−	−	−	−	18 − 23 (20)	−	−	−	20 − 22
shaft length (VS)	VAF	−	−	14 − 18	14 − 20 (17)	−	−	−	17 − 20
point length (VP)	VAE	9	9 − 10	8 − 10	8 − 11 (10)	8 − 9	8.2	9 − 10	8 − 9
proximal part inner length (VIP)	−	−	−	−	15 − 31 (24)	−	−	−	24 − 28
proximal part outer length (VOP)	−	−	19	−	16 − 24 (19)	−	−	−	20 − 24
span between roots (VSR)	−	−	23	−	16 − 27 (21)	−	−	−	17 − 21
inner root length (VIR)	VAD	16 − 19	16 − 20	17 − 19	14 − 27 (18)	15 − 17	15.3	15 − 20	15 − 18
outer root length (VOR)	VAC	8 − 9	8 − 11	8 − 10	7 − 15 (10)	12 − 13	12	11 − 15	11 − 15
Ratio VIR to VAC				1.7 − 2.1	1.2 − 2.3 (1.77)			1.2 − 1.6	
DORSAL ANCHOR									
inner length (DI)	DAA	28 − 40	37 − 41	39 − 42	37 − 43 (40)	34 − 36	33.9	30 − 39	32 − 38
main part length (DM)	DAB	24 − 27	25 − 29	27 − 29	26 − 32 (29)	24 − 26	24.6	24 − 28	24 − 28
distal part length (DD)	−	−	−	−	18 − 23 (21)	−	−	−	19 − 22
shaft length (DS)	DAF	−	−	17 − 22	15 − 21 (18)	−	−	−	16 − 20
point length (DP)	DAE	9	8 − 11	9 − 10	7 − 12 (9)	7 − 8	7.8	9 − 10	9 − 10
proximal part inner length (DIP)	−	−	28	−	26 − 30 (28)	−	−	−	22 − 27
proximal part outer length (DOP)	−	−	−	−	16 − 21 (19)	−		−	15 − 20
span between roots (DSR)	−	−	−	−	16 − 29 (23)	−		−	14 − 19
inner root length (DIR)	DAD	16 − 18	16 − 18	17 − 19	15 − 27 (20)	15 − 18	15.8	13 − 19	11 − 17
outer root length (DOR)	DAC	7 − 9	8 − 10	9 − 10	8 − 11 (9)	8 − 10	8.8	8 − 11	8 − 10
MARGINAL HOOK									
Total lenght	HTL	9 − 12	12 − 13	11 − 13	11 − 14 (12)	−	−	11 − 13	11 − 12
Sickel lenght	−	−	−	−	4 − 7 (5)	−	−	−	5 − 6
Shaft lenght	−	−	−	−	6 − 8 (7)	−	−	−	6 − 7
Philamentous	−	−	−	−	4 − 4 (4)	−	−	−	−
VENTRAL BAR									
height (VBH)	−	−	−	−	10 − 15 (13)	−	−	−	7 − 11
width (VBW)	VBL	31 − 43	37 − 57	37 − 43	54 − 66 (59)	40 − 42	40.8	36 − 47	36 − 46
span between processes (VBS)	VBDP	8 − 9	6 − 13	5 − 11	11 − 12 (12)	−	−	3 − 9	2 − 5
DORSAL BAR									
height (DBH)	−	−	−	−	6 − 9 (7)	−		−	4 − 6
width (DBW)	DBL	32 − 37	32 − 51	32 − 39	53 − 69 (60)	38 − 40	38.4	37 − 45	37 − 46
COPULATORY ORGAN									
length (CTL)	COL	38 − 88	73 − 92	73 − 85	104 − 116 (110)	80 − 90	−	79 − 103	85 − 98
ACCESSORY PIECE OF COPULATORY ORGAN									
length (APL)	APTL	25 − 28	27 − 33	27 − 33	40	30	−	26 − 32	23 − 34
width (APW)	−	−	−	−	19 − 57 (30)	−	−	−	5 − 8
upper lobe length (APUL)	−	−	−	−	21 − 21 (21)	−	−	−	15 − 18
lower lobe length (APLL)	APSL	−	−	22 − 23*	8 − 8 (8)	−	−	−	4 − 6
span between tips of upper and lower lobes (APPS)	−	−	−	−	15	−	−	−	8 − 13
VAGINA									
length (VL)	VL	29 − 35	29 − 69	35 − 51	54 − 90 (70)	40 − 45	−	25 − 60	45 − 60

* Corrected measurement from Fig. 7h in [28]: Approximately 10 micrometers. ** Abbreviation from Sarabeev et al. [29]. Mean in parentheses

Phylogenetic analyses were performed using Maximum Likelihood (ML) and Bayesian Inference (BI) methods. ML was carried out with RAxML version 7.0.4 [20] and BI analyses were run with MrBayes version 3.2.7 [21] using the online interface CIPRES (Cyberinfrastructure for Phylogenetic Research) Science Gateway v3.3 [22]. The best substitution model was estimated with the Akaike information criterion (AIC) using the jModel Test version 0.1.1 program [23], which predicted the best model for the 28S dataset to be GTR + I + G and for the ITS1 dataset, GTR + G. Nodal ML support was achieved through 1,000 bootstrap replicates. Bayesian analyses were performed using 10,000,000 generations with two independent runs, with sampling every 1,000 generations, a heating parameter value of 0.2, and the first 25% of the sampled trees were discarded. The significance of the phylogenetic relationships was estimated using posterior probabilities and bootstrap. Trees were drawn using FigTree program v.1.4.4 [24]. Uncorrected P distances were obtained in MEGA11 [25].

## Results

### Morphological Analysis

The specimens of *Ligophorus* were identified as *L. mediterraneus* parasitizing *M.cephalus* and *L. yucatanensis* parasitizing *M. curema*. The identification of both species was accomplished by studying the details of the male copulatory organ (MCO). In the first case, *L. mediterraneus* possess an accessory piece with an inwardly curved upper branch (Fig. 4C), whereas *L. yucatanensis* exhibits a claw-shaped accessory piece, a tunneled main lobe, and a thick-walled bulb-shaped opening (Fig. 4G; Table 4).

**Table 4:** Comparative metrical data for *Ligophorus yucatanensis*

Species		*L. yucatanensis*	*L. yucatanensis*
Reference		Present study	[2]
Locality		Off Ría Lagartos, Yucatán	Celestun Lagoon, Yucatán
Host		*Mugil curema*	*Mugil cephalus*
n	**	11	10
VENTRAL ANCHOR			
inner length (VI)	VAA	28 − 35 (30)	29 − 33
main part length (VM)	VAB	16 − 22 (19)	17 − 21
distal part length (VD)	−	13 − 18 (15)	−
shaft length (VS)	VAF	9 − 14 (12)	12 − 14
point length (VP)	VAE	8 − 12 (9)	7 − 10
proximal part inner length (VIP)	−	21 − 28 (25)	−
proximal part outer length (VOP)	−	12 − 18 (15)	−
span between roots (VSR)	−	15 − 23 (18)	−
inner root length (VIR)	VAD	12 − 18 (15)	17 − 20
outer root length (VOR)	VAC	4 − 8 (7)	5 − 10
DORSAL ANCHOR			
inner length (DI)	DAA	30 − 39 (33)	37 − 40
main part length (DM)	DAB	23 − 28 (24)	20 − 28
distal part length of (DD)	−	13 − 20 (17)	−
shaft length (DS)	DAF	12 − 16 (14)	15 − 20
point length (DP)	DAE	7 − 11 (10)	8 − 9
proximal part inner length (DIP)	−	21 − 29 (24)	−
proximal part outer length (DOP)	−	14 − 19 (17)	−
span between roots (DSR)	−	12 − 21 (16)	−
inner root length (DIR)	DAD	11 − 19 (14)	16 − 20
outer root length (DOR)	DAC	6 − 14 (8)	5 − 12
MARGINAL HOOK			
Total lenght	HTL	10 − 14 (12)	9 − 13
Sickel lenght	−	4 − 6 (5)	−
Shaft lenght	−	5 − 8 (7)	−
Philamentous	−	5 − 6 (6)	−
VENTRAL BAR			
height (VBH)	−	6 − 13 (9)	−
width (VBW)	VBL	39 − 60 (51)	39 − 47
span between processes (VBS)	VBDP	9 − 13 (11)	5 − 8
DORSAL BAR			
height (DBH)	−	5 − 10 (8)	−
width (DBW)	DBL	49 − 61 (54)	36 − 47
COPULATORY ORGAN			
length (CTL)	COL	89 − 113 (103)	75 − 103
ACCESSORY PIECE OF COPULATORY ORGAN			
length (APL)	APTL	26 − 35 (32)	24.5 − 29.2
width (APW)	−	14 − 35 (23)	−
upper lobe length (APUL)	−	14 − 18 (15)	−
lower lobe length (APLL)	APSL	3 − 10 (7)	3 − 9
span between tips of upper and lower lobes (APPS)	−	8 − 14 (11)	−
VAGINA			
length (VL)	VL	42 − 67 (56)	23 − 41

** Abbreviation from Sarabeev et al. [29]. Mean in parentheses

### Molecular Data and Phylogenetic Analyses

**Fig. 2:**
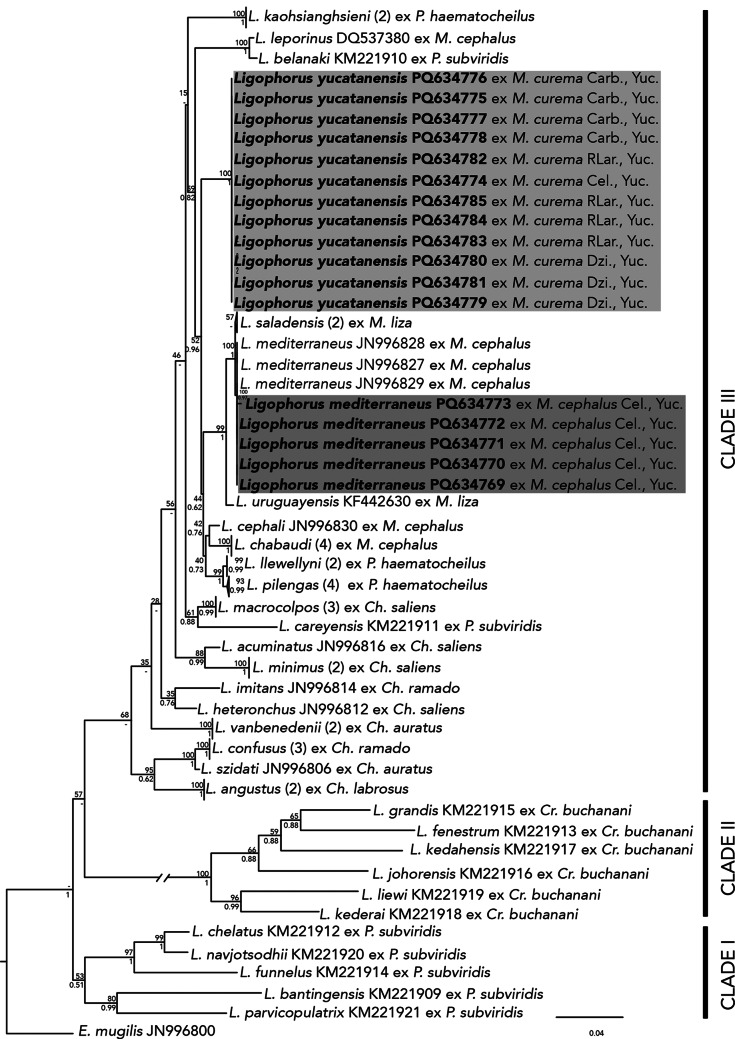
Consensus Bayesian inference (BI) tree and Maximum likelihood (ML) tree inferred from the 28S gene from nuclear ribosomal DNA. Numbers on internal nodes show posterior probabilities (BI) and ML bootstrap clade frequencies. Sequences generated in this study are in bold. The number of available sequences in GenBank is noted next to each species

The 28S phylogenetic analyses inferred with ML and BI recovered similar topologies (Fig. 2). The analyses showed that *Ligophorus* is monophyletic and included three major clades with high posterior probabilities support values (Fig. 2). Clade I is formed by five species of *Ligophorus* parasitizing *P. subviridis* albeit with low bootstrap and posterior probability support values (53/0.51). Clade II is formed by six species of *Ligophorus* parasitizing *Cr. buchanani* (100/1). Clade III is formed by the remaining 21 species of *Ligophorus* parasitizing different host species including members of *Mugil*, *Chelon*, and *Planiliza* (68/-). The 17 newly sequenced individuals from two host species were nested in Clade III, however their position on the tree showed that we sequenced specimens from two separate species (Fig. 2).

The five newly sequenced isolates collected from *M. cephalus* in Celestún, Yucatán were nested with sequences of *L. mediterraneus* (GenBank JN996827–29) from *M. cephalus* off Cullera Spain, showing they were conspecific, with high nodal support (100/0.97). Furthermore, the 12 newly sequenced isolates of *L. yucatanensis* collected from *M*. *curema* in four coastal lagoons of Yucatán formed an independent group also highly supported (1/100). These 12 isolates were recovered as the sister group of two subclades, one formed by (*L. llewellyni* + *L. pilengas*) *+* (*L. cephali + L. chabaudi*); and one by *L. uruguayensis* (*L. saladensis* + *L. mediterraneus*), with moderate to high support (52/0.96) (Fig. 2).

**Fig. 3:**
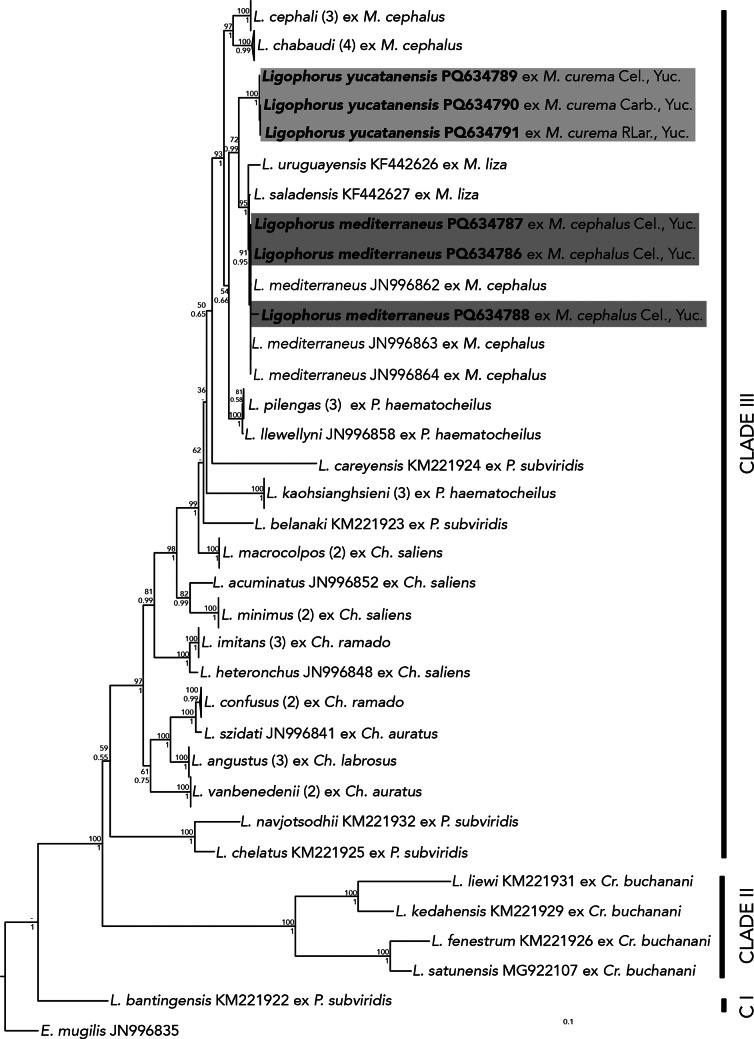
Consensus Bayesian inference (BI) tree and Maximum likelihood (ML) tree inferred from the internal transcribed spacer 1 from nuclear ribosomal DNA. Numbers on internal nodes show posterior probabilities (BI) and ML bootstrap clade frequencies. Sequences generated in this study are in bold. The number of available sequences in GenBank is noted next to each species

The ITS1 phylogenetic analyses inferred with ML and BI also recovered similar topologies (Fig. 3). The tree yielded *Ligophorus* as a monophyletic assemblage with high posterior probabilities support value. The three new ITS1 isolates from *L*. *mediterraneus* were nested with three sequences identified as *L. mediterraneus* (JN996862–64), from *M. cephalus* off Cullera Spain, with strong support (91/0.95). Furthermore, the three newly sequenced isolates of *L. yucatanensis* also formed an independent and highly supported clade (1/100). However, in this tree, this clade was recovered as the sister group of *L. uruguayensis* (*L. saladensis* + *L. mediterraneus*), with moderate to high nodal support (72/0.99).

The intraspecific genetic divergence among the five sequences of *L. mediterraneus* was null, whereas the divergence between these individuals and those from the Mediterranean (GenBank JN996827–29) varied from 0 to 0.2% for 28S, and 0–0.1% for ITS1. Moreover, the intraspecific genetic divergence among the specimens of *L. yucatanensis* was null for the 28S, and varied from 0 to 0.2% for ITS1 (see Supplementary Table S1). The divergence between the two species of *Ligophorus* from the Yucatán Peninsula was 3.4–5.9% for 28S, and 6.5–8% for ITS1.

## Discussion

In this study, we identified two species of *Ligophorus* parasitizing mugilids from the northern coast of the Yucatán Peninsula based on a combination of morphological and molecular characters, namely *L. mediterraneus* and *L. yucatanensis*. Regarding *L. mediterraneus*, this species is difficult to distinguish from *L. mugilinus* based on morphological and morphometric characters since most of them are overlapped. The taxonomic history between these two species is rather controversial [26–29].

For instance, Hargis [30] described *Pseudohaliotrema mugilinus* from *M. cephalus* in Alligator Harbor, Florida, USA, in the Gulf of Mexico (GoM). Euzet and Suriano [31] erected the genus *Ligophorus* and transferred the species of Hargis [30] as *L. mugilinus.* In addition, after examining specimens of *Ligophorus* sampled from *M. cephalus* in the Mediterranean Sea, these authors concluded that those specimens corresponded to *L. mugilinus.* Later, Sarabeev et al. [26] described *Ligophorus mediterraneus* from *M. cephalus* off the Western coast of the Mediterranean Sea, and redescribed *L. mugilinus* based on specimens sampled from South Carolina, in the Northwest Atlantic (GoM). These authors further differentiated both species by using three main characters, i.e., (1) a V-shaped dorsal bar in *L. mugilinus*, and slightly bowed in *L. mediterraneus*; (2) a well-developed sclerotized median process of the ventral bar in *L. mugilinus*, whereas the process is absent in *L. mediterraneus*; and (3) a straight distal end of the MCO in *L. mugilinus* and curved in *L. mediterraneus*. Sarabeev et al. [26] concluded that the specimens of *L. mugilinus* reported by Euzet and Suriano [31] only from the Mediterranean, corresponded in fact with *L. mediterraneus.* Therefore, according to Sarabeev et al. [26], *L. mugilinus* was distributed in GoM and *L. mediterraneus* in the Mediterranean and Black Seas [26]. More recently, Saraveeb et al. [28] conducted a comprehensive morphological study of the genus *Ligophorus* and highlighted the shape of the secondary lobe of the accessory piece of the MCO as the main character for distinguishing between both species. In *L. mugilinus*, the lobe is straight or backwardly curved, whereas in *L. mediterraneus* the lobe is inwardly curved, which is consistent with the assessment by Dmitrieva et al. [27] who redescribed *L. mediterraneus* based on 20 specimens sampled from *M. cephalus* from the Black Sea and five from the Mediterranean and proposed some additional characters to distinguish both species.

**Fig. 4:**
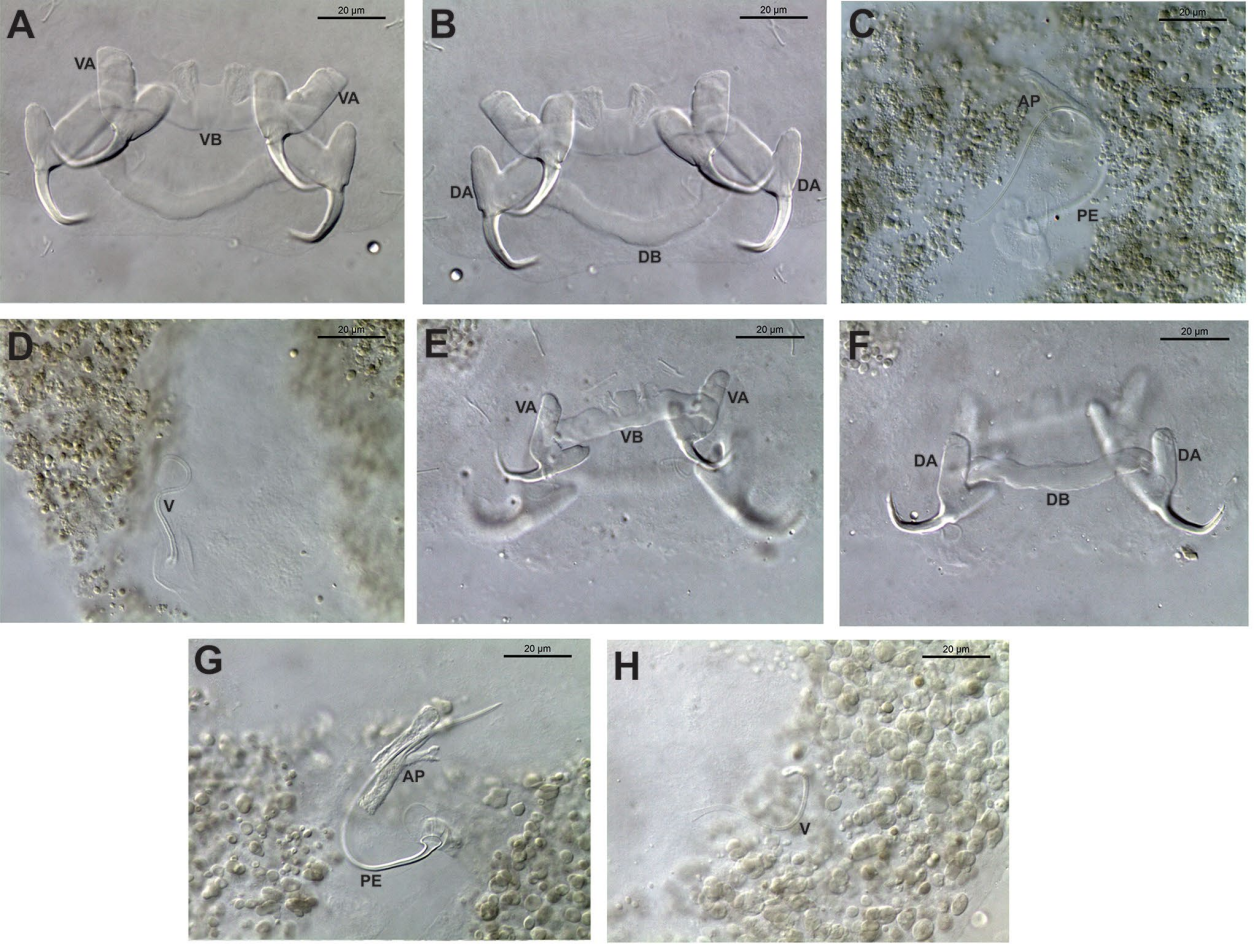
Photomicrographs of sclerotized elements of haptor and male copulatory complex of *Ligophorus mediterraneus* (**A**–**D**) and *L. yucatanensis* (**E**–**H**). (**A**, **E**) Ventral bar VB and anchors VA. (**B**, **F**) Dorsal bar DB and anchors DA. (**C**, **G**) Male copulatory complex MCO, highlighting the penis PE and accessory piece AP. (**D**, **H**) Vaginal armament V

The specimens sampled from *M. cephalus* in Celestún, Yucatán morphologically correspond with *L. mediterraneus* because of the presence of a slightly inwardly curved lobe of the accessory piece. Still, it would be necessary to obtain sequences of *L. mugilinus* from their type-host and locality to corroborate the interrelationships between these two species, which now ocurr sympatrically in the GoM; the results of the present study show that *L. mediterraneus* is also distributed in that geographical area. A sequence of the 28S rRNA gene of *L. mugilinus* is available in GenBank (AF131710); however this sequence is very short (374 bp long) and does not contribute to resolve the species delimitation between these two species, once the alignment is trimmed for the shortest sequence, a large polytomy is yielded (tree not shown). In our phylogenetic tree, *Ligophorus mediterraneus* was recovered as the sister species of *L. saladensis*, a species reported as a parasite of *Mugil liza* Günther, off the coast of Buenos Aires, Argentina [32]. They are also very similar morphologically because both posses a bilobed accessory piece of the MCO, with the lower lobe smaller than the upper, and a ventral bar with a medial process [4]; however, they can also be differentiated by morphology of the distal end of the accessory piece of the MCO. In *L. saladensis*, as in *L. mugilinus*, the secondary lobe of the accessory piece is forwardly directed; furthermore, the size of some morphological traits of *L. saladensis* are different. For instance, the ventral anchor point, the marginal hooklets and the accessory piece are shorter in *L. saladensis* than in *L. mediterraneus*, and the ventral bar and the vagina are longer [32].

Additionally, we sequenced specimens of *Ligophorus yucatanensis* for the first time. The species was originally described by Rodríguez-Gónzalez et al. [3] as a parasite of *M. cephalus* in Celestún Lagoon, Yucatán, Mexico, but no sequence data were provided. The species is easily differentiated from all congeners by having a claw-shaped accessory piece of the MCO, as clearly seen on Figs. 2 and 3A in Rodríguez-Gónzalez et al. [3]. Except by having a slightly overall smaller size in some sclerotized structures (see comparison in Table 4), our specimens collected from *Mugil curema* in four coastal lagoons of Northern Yucatán Peninsula correspond entirely with *L. yucatanensis*, because they possess a claw-shaped accessory piece of the MCO (Fig. 4G). The presence of *L. yucatanensis* in *M. curema* represents a new host record. Apparently, this species only occurs in coastal lagoons since it has not been found in species of mugilids occurring offshore, and can be considered as endemic to the coastal lagoons of Yucatán.

Regarding the topology of the ITS1 phylogenetic tree, it yielded *L. yucatanensis* as the sister species of a clade containing *L. uruguayensis*,* L. saladensis*, and *L. mediterraneus*, and this agrees with the study of Acosta et al. [9] which considers *L. uruguayensis*,* L. saladensis*, and *L. mediterraneus* occurring across the south-western and north-eastern Atlantic Ocean. In fact, some authors have previously discussed the possibility that *L. uruguayensis* and *L. saladensis* represent a species complex along with *L. mediterraneus* due to low genetic divergence values among them. Marchiori et al. [5] reported that the genetic divergence between *L. mediterraneus* and *L. saladensis* was 0.1–0.2%, and 0.4–0.6%, for the 28S and ITS1, respectively. Our findings also suggest that *L. mediterraneus* and *L. saladensis* could represent the same genetic lineage (Table S1), although morphological differences separate these species. More information is needed to resolve species limits and the phylogenetic interrelationships among members of this clade of *Ligophorus*. We suggest sequencing the mitochondrial gene COI, a more variable molecular marker, to further elucidate the genetic variation among the species forming the clade composed by *L. mediterraneus*,* L. salandensis* and *L. uruguayensis*.

## Electronic Supplementary Material

Below is the link to the electronic supplementary material.

Supplementary Material 1

## Data Availability

No datasets were generated or analysed during the current study.
