# Supplementary material for: Morphological and Molecular Characterization of Two Species of Ligophorus (Monogenea: Ancyrocephalidae) in Mullets from the Yucatán Peninsula, with Comments on the Geographical Distribution of L. mediterraneus
Source: Acta Parasitol. 2025 Jan 24;70(1):30. doi: 10.1007/s11686-024-00953-5 (PMC11761990; doi:10.1007/s11686-024-00953-5)
Supplement: Supplementary file 1 — Supplementary Material 1 [file 11686_2024_953_MOESM1_ESM.docx]

Table S1. Pairwise nucleotide sequence comparisons between taxa for the aligned 28S rDNA sequences (1,007 nt) and for ITS1 sequences (892 nt). In bold is represented the genetic intraspecific divergence.

|  | **28S** | 1 | 2 | 3 | 4 | 5 | 6 | 7 | 8 | 9 | 10 | 11 | 12 | 13 | 14 | 15 | 16 | 17 | 18 | 19 | 20 | 21 | 22 | 23 | 24 | 25 | 26 | 27 | 28 | 29 | 30 | 31 | 32 |
| --- | --- | --- | --- | --- | --- | --- | --- | --- | --- | --- | --- | --- | --- | --- | --- | --- | --- | --- | --- | --- | --- | --- | --- | --- | --- | --- | --- | --- | --- | --- | --- | --- | --- |
| 1 | *L. parvicopulatrix* | – | – | – | – | – | – | – |  |  |  |  |  |  |  |  |  |  |  |  |  |  |  |  |  |  |  |  |  |  |  |  |  |
| 2 | *L. bantingensis* | 10.9 | – | – | – | – | – | – |  |  |  |  |  |  |  |  |  |  |  |  |  |  |  |  |  |  |  |  |  |  |  |  |  |
| 3 | *L. funnelus* | 12.1 | 11.7 | – | – | – | – | – |  |  |  |  |  |  |  |  |  |  |  |  |  |  |  |  |  |  |  |  |  |  |  |  |  |
| 4 | *L. chelatus* | 10.1 | 11.8 | 7.3 | – | – | – | – |  |  |  |  |  |  |  |  |  |  |  |  |  |  |  |  |  |  |  |  |  |  |  |  |  |
| 5 | *L. navjotsodhii* | 10.1 | 11.7 | 7.4 | 2.6 | – | – | – |  |  |  |  |  |  |  |  |  |  |  |  |  |  |  |  |  |  |  |  |  |  |  |  |  |
| 6 | *L. kederai* | 17.5 | 18.8 | 17.6 | 17 | 16.3 | – | – |  |  |  |  |  |  |  |  |  |  |  |  |  |  |  |  |  |  |  |  |  |  |  |  |  |
| 7 | *L. liewi* | 18 | 20.3 | 19 | 18.3 | 17.3 | 8.7 | – |  |  |  |  |  |  |  |  |  |  |  |  |  |  |  |  |  |  |  |  |  |  |  |  |  |
| 8 | *L. johorensis* | 18.6 | 19.5 | 17.7 | 17.6 | 17.4 | 10.8 | 11 | – |  |  |  |  |  |  |  |  |  |  |  |  |  |  |  |  |  |  |  |  |  |  |  |  |
| 9 | *L. kedahensis* | 17.8 | 17.8 | 17.8 | 18.3 | 17.9 | 12.2 | 13.2 | 10.2 | – |  |  |  |  |  |  |  |  |  |  |  |  |  |  |  |  |  |  |  |  |  |  |  |
| 10 | *L. fenestrum* | 18.1 | 19.6 | 17.7 | 18.2 | 17.9 | 11 | 12.4 | 11.1 | 10.3 | – |  |  |  |  |  |  |  |  |  |  |  |  |  |  |  |  |  |  |  |  |  |  |
| 11 | *L. grandis* | 20.1 | 19.9 | 19.4 | 19.8 | 20.1 | 11.1 | 12.1 | 9.2 | 9.4 | 8.5 | – |  |  |  |  |  |  |  |  |  |  |  |  |  |  |  |  |  |  |  |  |  |
| 12 | *L. angustus* | 12.3–12.7 | 12.1 | 13.4–13.8 | 11.8–12.2 | 12–12.4 | 17.4–17.8 | 18.6–18.7 | 17.9–18.3 | 17.1–17.2 | 19–19.4 | 19.2–19.4 | **0.11** |  |  |  |  |  |  |  |  |  |  |  |  |  |  |  |  |  |  |  |  |
| 13 | *L. szidati* | 11.6 | 11.5 | 11.9 | 10.9 | 11.3 | 18.3 | 19.1 | 19 | 17.3 | 19.3 | 19.3 | 4.4 | – |  |  |  |  |  |  |  |  |  |  |  |  |  |  |  |  |  |  |  |
| 14 | *L. confusus* | 12.2 | 12 | 12.2 | 10.9 | 11 | 18.2 | 19 | 19 | 17.7 | 19.8 | 19.3 | 4.8–5.1 | 1–1.1 | **0** |  |  |  |  |  |  |  |  |  |  |  |  |  |  |  |  |  |  |
| 15 | *L. vanbenedenii* | 11.9 | 11.9 | 12.8 | 11.2 | 10.6 | 17.4 | 18.8 | 18.4 | 16.4 | 18.4 | 19.4 | 6.6–6.7 | 6.4 | 6.9–7.1 | **0** |  |  |  |  |  |  |  |  |  |  |  |  |  |  |  |  |  |
| 16 | *L. imitans* | 11.2 | 11.6 | 10.6 | 9.7 | 8.3 | 16.6 | 17.7 | 16.4 | 15.1 | 17.6 | 18.1 | 6.6–6.7 | 6.5 | 6.5 | 6.7 | – |  |  |  |  |  |  |  |  |  |  |  |  |  |  |  |  |
| 17 | *L. heteronchus* | 12.3 | 12.4 | 11.5 | 10.7 | 9.7 | 17.2 | 18.4 | 17.3 | 15.4 | 18 | 18.6 | 6.1–6.2 | 5.9 | 6.1–6.5 | 5 | 3.5 | – |  |  |  |  |  |  |  |  |  |  |  |  |  |  |  |
| 18 | *L. acuminatus* | 10.6 | 12.2 | 10.8 | 10.7 | 9.7 | 16 | 17.5 | 17 | 15.7 | 16.7 | 18.5 | 6.4–6.7 | 6.1 | 6.5–6.9 | 6 | 4.8 | 4.9 | – |  |  |  |  |  |  |  |  |  |  |  |  |  |  |
| 19 | *L. minimus* | 10.6 | 11.9–12 | 12 | 10.9 | 9.6 | 16.8–16.9 | 18.6 | 17.8 | 15.9–16 | 17.8 | 19.4 | 7.9–8.4 | 7.3–7.9 | 8.3 | 7.3–7.5 | 5.4–5.6 | 5.6–5.9 | 3.1–3.3 | **0** |  |  |  |  |  |  |  |  |  |  |  |  |  |
| 20 | *L. careyensis* | 12.5 | 12.9 | 11.5 | 10.7 | 10.1 | 15.7 | 18.1 | 16.7 | 16.7 | 17.1 | 17.9 | 9.5–9.9 | 9.2 | 9.3 | 9.4 | 7.6 | 7.2 | 6.9 | 8.3 | – |  |  |  |  |  |  |  |  |  |  |  |  |
| 21 | *L. macrocolpos* | 11.3 | 11.6 | 10.2 | 10 | 9 | 17.4 | 18.2 | 17 | 16.4 | 17.8 | 19.4 | 6.8–7.2 | 6.2–6.4 | 6.7–7 | 5.7 | 4.5 | 4.4–4.6 | 3.9–4 | 5–5.1 | 5.5 | **0** |  |  |  |  |  |  |  |  |  |  |  |
| 22 | *L. kaohsianghsieni* | 12.7–12.8 | 13–13.3 | 11.7–11.8 | 11.2–11.3 | 10.5–10.6 | 17.3–17.4 | 17.6–17.7 | 17 | 16.3 | 17.1–17.3 | 18.7 | 8–8.1 | 7.5–7.6 | 7.8–7.9 | 6.8–6.9 | 6.2–6.3 | 5.7–5.8 | 5 | 6.4–6.5 | 7 | 4.2 | **0** |  |  |  |  |  |  |  |  |  |  |
| 23 | *L. belanaki* | 12 | 13.6 | 11.6 | 10.2 | 9.8 | 16.1 | 18.3 | 17 | 16.8 | 17.7 | 18.7 | 8.8–9 | 8.6 | 8.9 | 8.5 | 7.7 | 7.2 | 6.5 | 7.3 | 6.7 | 5.2 | 6.1 | – |  |  |  |  |  |  |  |  |  |
| 24 | *L. leporinus* | 11.6 | 13.2 | 11.6 | 10.4 | 9.9 | 16.3 | 18.1 | 16.9 | 16.8 | 17.6 | 18.4 | 8.5–8.7 | 8.2 | 8.5 | 7.9 | 7.2 | 6.5 | 6.4 | 7.2 | 6.7 | 4.9 | 5.6 | 0.65 | – |  |  |  |  |  |  |  |  |
| 25 | *L. yucatanensis* | 11.8–18.8 | 12–16.7 | 10.9–17.7 | 10–16.3 | 9.2–14.1 | 15.7–24.7 | 17.4–25.7 | 15.5–22.4 | 14.8–19.4 | 17–23.4 | 17.7–23.6 | 7.4–9.9 | 7–8.8 | 7.3–9.7 | 6.5–8.5 | 5.6–7.8 | 5.1–6.9 | 5–6.9 | 6.1–8.6 | 6.6–12 | 4.3–6.3 | 4.7–6.8 | 4.5–8.2 | 4.2–7.6 | **0** |  |  |  |  |  |  |  |
| 26 | *L. llewellyni* | 11.9 | 12.3 | 11.5 | 9.9 | 9.4 | 16 | 18 | 16.6 | 15.5 | 17.6 | 18.1 | 7.2–7.5 | 6.9 | 7.2 | 6–6.2 | 5.6 | 4.2–5.3 | 4.6 | 6.2 | 6.7 | 3.6 | 4.8 | 4.6 | 4.4 | 2.8–4.2 | 0 |  |  |  |  |  |  |
| 27 | *L. pilengas* | 11.9–12.05 | 12.1–12.3 | 11.3–11.5 | 9.7–9.9 | 9.1–9.3 | 16.3–16.4 | 18–18.2 | 16.7–16.8 | 15.5–15.7 | 17.9–18.1 | 18.2–18.4 | 6.8–7.2 | 6.5–6.7 | 6.8–7.1 | 5.6–5.8 | 5.6–5.7 | 4.9–5.1 | 4.5–4.7 | 6.3 | 6.7–6.9 | 3.3–3.6 | 4.8–4.9 | 4.9–5 | 4.6–4.8 | 2.9–4.3 | 0.5–0.6 | 0.1 |  |  |  |  |  |
| 28 | *L. cephali* | 11.3 | 11.6 | 10.6 | 9.6 | 8.9 | 16 | 17.8 | 16.6 | 15.5 | 17.8 | 18.8 | 6.6 | 6 | 6.6–6.8 | 5.5 | 5.1 | 4.6 | 4.4 | 5.6 | 6.3 | 2.7 | 4.1 | 4.3 | 4.2 | 2.8–4 | 1.7 | 1.8–1.9 | – |  |  |  |  |
| 29 | *L. chabaudi* | 11.5 | 11.6 | 9.9 | 9.2 | 8.6 | 16.3 | 17.6 | 16.2 | 15.4 | 17.4 | 18.1 | 6.3–6.4 | 6.2 | 6.5–6.9 | 5.9 | 5 | 4.3 | 4.8 | 6–6.1 | 6.7 | 3.7 | 4.9 | 5.2 | 5 | 3.3–4.9 | 2.3–2.4 | 2.4–2.6 | 1.8 | **0** |  |  |  |
| 30 | *L. uruguayensis* | 11.3 | 11.3 | 10.7 | 9.6 | 8.7 | 15.8 | 17.4 | 16.5 | 15.3 | 17.8 | 18.4 | 7 | 6.6 | 6.9–7.3 | 6.5 | 5 | 4.9 | 4.6 | 6–6.1 | 6.5 | 3.1–3.2 | 4.9 | 5.4 | 5.2 | 3.3–5 | 2.9–3 | 2.9–3 | 2 | 2.8 | – |  |  |
| 31 | *L. saladensis* | 11.1–11.3 | 11.2–11.3 | 11.1–11.3 | 9.7–9.9 | 9–9.2 | 15.7 | 17.7 | 16.6 | 15.4 | 17.7 | 18.2 | 7.1–7.2 | 6.8–6.9 | 7.4–7.8 | 6.3–6.4 | 5.3–5.4 | 5.5–5.6 | 4.7–4.8 | 6.1–6.2 | 6.9–7.1 | 3.5–3.7 | 5.1–5.2 | 5.7–5.8 | 5.5–5.6 | 3.4–5.3 | 3–3.1 | 2.8–3 | 2.1–2.2 | 3.1–3.2 | 0.9–1 | **0.1** |  |
| 32 | *L. mediterraneus* | 10.8–11.3 | 10.8–11.4 | 10.8–11.6 | 9.5–10.3 | 8.8–9.5 | 15.1 | 17.2 | 16 | 14.9 | 17.2 | 17.6 | 7.1–7.6 | 6.8–7.3 | 7.4–7.8 | 6.4–6.6 | 5.4–5.6 | 5.5–6 | 4.9–5.1 | 6.2–6.4 | 6.6–7.3 | 3.4–3.6 | 4.9–5.3 | 5.5–6 | 5.3–5.8 | 3.4–5.9 | 3–3.1 | 2.8–3.1 | 2–2.2 | 3.1–3.5 | 1–1.3 | 0.1–0.4 | 0–0.2 |

|  | **ITS1** | 1 | 2 | 3 | 4 | 5 | 6 | 7 | 8 | 9 | 10 | 11 | 12 | 13 | 14 | 15 | 16 | 17 | 18 | 19 | 20 | 21 | 22 | 23 | 24 | 25 | 26 | 27 |
| --- | --- | --- | --- | --- | --- | --- | --- | --- | --- | --- | --- | --- | --- | --- | --- | --- | --- | --- | --- | --- | --- | --- | --- | --- | --- | --- | --- | --- |
| 1 | *L. bantingensis* | – | – | – | – |  |  |  |  |  |  |  |  |  |  |  |  |  |  |  |  |  |  |  |  |  |  |  |
| 2 | *L satunensis* | 42.3 | – | – | – |  |  |  |  |  |  |  |  |  |  |  |  |  |  |  |  |  |  |  |  |  |  |  |
| 3 | *L. fenestrum* | 41.3 | 11.8 | – | – |  |  |  |  |  |  |  |  |  |  |  |  |  |  |  |  |  |  |  |  |  |  |  |
| 4 | *L. kedahensis* | 36.8 | 28.7 | 27.2 | – |  |  |  |  |  |  |  |  |  |  |  |  |  |  |  |  |  |  |  |  |  |  |  |
| 5 | *L. liewi* | 43.8 | 31.5 | 34 | 20 | – |  |  |  |  |  |  |  |  |  |  |  |  |  |  |  |  |  |  |  |  |  |  |
| 6 | *L. chelatus* | 29.3 | 40.6 | 40.9 | 38.4 | 43.1 | – |  |  |  |  |  |  |  |  |  |  |  |  |  |  |  |  |  |  |  |  |  |
| 7 | *L. navjotsodhii* | 30.1 | 41.2 | 39.3 | 37.3 | 41.3 | 11.7 | – |  |  |  |  |  |  |  |  |  |  |  |  |  |  |  |  |  |  |  |  |
| 8 | *L. vanbenedenii* | 28 | 37.1 | 39.3 | 36.3 | 39.4 | 24.4 | 27.4 | **0** |  |  |  |  |  |  |  |  |  |  |  |  |  |  |  |  |  |  |  |
| 9 | *L. angustus* | 29 | 36.6 | 39.8 | 38.5 | 41.1 | 24.2 | 27.2 | 13.1 | **0** |  |  |  |  |  |  |  |  |  |  |  |  |  |  |  |  |  |  |
| 10 | *L. szidati* | 27.9 | 38.4 | 39.7 | 37.6 | 40.2 | 24.8 | 27.3 | 14 | 9.7 | – |  |  |  |  |  |  |  |  |  |  |  |  |  |  |  |  |  |
| 11 | *L. confusus* | 27.8–28 | 38–38.3 | 39.6–40 | 38.3–38.6 | 40.8–41.6 | 25 | 27.1 | 14.1 | 9.1–9.4 | 2–2.3 | **0.29** |  |  |  |  |  |  |  |  |  |  |  |  |  |  |  |  |
| 12 | *L. heteronchus* | 30 | 39.3 | 41.6 | 39 | 43 | 25 | 27.6 | 14.7 | 13.7 | 15.6 | 15.4 | – |  |  |  |  |  |  |  |  |  |  |  |  |  |  |  |
| 13 | *L. imitans* | 30.7 | 39.6 | 41.8 | 39.5 | 42.9 | 25.2 | 26.7 | 15.3 | 14.5 | 16 | 15.5–15.7 | 3.4 | **0** |  |  |  |  |  |  |  |  |  |  |  |  |  |  |
| 14 | *L. acuminatus* | 29.5 | 46.8 | 46.1 | 44.1 | 48.2 | 27.9 | 31.2 | 18.5 | 19 | 22.9 | 21.9 | 16 | 17.2 | – |  |  |  |  |  |  |  |  |  |  |  |  |  |
| 15 | *L. minimus* | 30.3 | 38.7 | 39.2 | 37 | 40.8 | 25.1 | 28.1 | 17.8 | 16.8 | 18.7 | 17.5–17.8 | 14.5 | 14.7 | 11 | **0** |  |  |  |  |  |  |  |  |  |  |  |  |
| 16 | *L. macrocolpos* | 28.9 | 39.2 | 40.5 | 37.2 | 40.7 | 25.7 | 27.2 | 16.6 | 17.7 | 19 | 18.3–18.6 | 14.6 | 14.5 | 14.4 | 14.7 | **0** |  |  |  |  |  |  |  |  |  |  |  |
| 17 | *L. belanaki* | 30.6 | 42.9 | 41.8 | 35.6 | 41.5 | 26 | 26.2 | 22 | 20.9 | 21.2 | 20.8–21.2 | 20.2 | 20.4 | 19.2 | 18.7 | 13 | – |  |  |  |  |  |  |  |  |  |  |
| 18 | *L. kaohsianghsieni* | 34.6–34.7 | 38.7–43.4 | 41.6–42 | 38.8–39.2 | 42.2–43.6 | 30.5–31 | 31.4–31.7 | 22–24.9 | 22–25.1 | 24–26.1 | 23.3–26.8 | 21.3–24.3 | 21.3–24.3 | 22.3–24.1 | 19.8–22.5 | 14 | 18.6–19.2 | **0** |  |  |  |  |  |  |  |  |  |
| 19 | *L. careyensis* | 31.2 | 42 | 39.9 | 38 | 43.6 | 28.1 | 28.8 | 25.9 | 27.2 | 28.2 | 28.2 | 24.4 | 25 | 28.1 | 23.4 | 20.5 | 22 | 23.5–23.6 | – |  |  |  |  |  |  |  |  |
| 20 | *L. cephali* | 30.2–31.2 | 40–43.1 | 40.8–41.7 | 36.9–38 | 40.1–40.5 | 25.9–26.8 | 26.8–27.6 | 19.6–21.9 | 21.2–23.7 | 22.2–23.8 | 21.7–24.4 | 19.4–21.6 | 19.3–21.6 | 17–18.9 | 14.7–16.5 | 12–13.4 | 14.5–15 | 16.2–18.4 | 21.2–21.8 | **0** |  |  |  |  |  |  |  |
| 21 | *L. chabaudi* | 31.2–36.1 | 39.6–43.8 | 40.9–44.2 | 36.8–40 | 39.3–40.2 | 27.5–30 | 28.4–31 | 19.1–22.7 | 20.1–24.1 | 21.1–24.6 | 19.8–24.2 | 19.3–22.2 | 18.8–21.5 | 19.5–22 | 15.9–17.3 | 11–13.2 | 14.7–16.3 | 16.3–18.6 | 20.7–22.9 | 7.6–8.7 | **0–0.6** |  |  |  |  |  |  |
| 22 | *L. pilengas* | 30–30.2 | 39.3–39.5 | 41.1–41.3 | 37.4 | 40.4–40.6 | 26.3–26.5 | 27.8–27.9 | 18.6–18.8 | 19.3–19.5 | 21–21.2 | 19.8–20.3 | 18.3–18.5 | 18.5–18.6 | 16.8 | 15.4–15.6 | 10–10.2 | 13.8–14 | 15.4–17.6 | 19.7–19.9 | 7.7–8.8 | 7.5–9 | 0–0.1 |  |  |  |  |  |
| 23 | *L. llewellyni* | 30.2 | 39.1 | 41 | 37.2 | 40.2 | 26.6 | 28.1 | 18.9 | 19.4 | 21 | 19.8–20.1 | 18.6 | 18.8 | 17 | 15.6 | 10.2 | 13.8 | 15.5–17.6 | 19.9 | 7.9–8.8 | 7.7–9 | 0.3–0.4 | – |  |  |  |  |
| 24 | *L. yucatanensis* | 30.2–31.5 | 43–43.3 | 42–42.1 | 38.3–38.5 | 41.9–42.3 | 26.8–27.7 | 27.3–28.3 | 20.4–20.9 | 21.6–22.1 | 22.7–23.1 | 22.2–23.1 | 21.5–22.1 | 21.3–22.2 | 18.9 | 17.5–18 | 14.5–15.4 | 17.3–18 | 19.4–21.1 | 20.6–21.1 | 11.6–12.3 | 11.6–12.2 | 7.9–8.5 | 7.9–8.3 | **0–0.2** |  |  |  |
| 25 | *L. uruguayensis* | 28.8 | 39.2 | 41.4 | 36.3 | 39.6 | 24.4 | 26 | 18 | 18.8 | 19.8 | 19.2–19.5 | 18.7 | 18.3 | 17.3 | 15.4 | 11.4 | 14.4 | 16.5–18.3 | 20 | 8.5–9.4 | 9.7–11.5 | 8.6–8.7 | 8.6 | 8.9–9.2 | – |  |  |
| 26 | *L. saladensis* | 29.6 | 38.9 | 40.5 | 36.6 | 40.1 | 24.6 | 25.8 | 18 | 18.5 | 19.5 | 18.9–19.2 | 17.8 | 17.2 | 15.9 | 14 | 10.7 | 13.6 | 15.2–16.8 | 19.2 | 7.5–8.3 | 8.7–10.3 | 6.5–6.7 | 6.5 | 6.4–6.6 | 2.8 | – |  |
| 27 | *L. mediterraneus* | 28.7–30.8 | 39–46 | 41.4–45.8 | 37.5–40.7 | 39.8–46.7 | 24.8–27.5 | 26.5–28 | 18.4–25.3 | 19–27 | 19.4–25 | 18.7–25.1 | 19–27 | 18–26 | 16–21 | 14–22 | 11–14 | 13.7–17.6 | 16–23 | 19–23 | 6.2–8.4 | 8.4–11.5 | 6.5–9.4 | 6.5–9 | 6.5–8 | 2.8–3.7 | 0.3–0.6 | 0–0.1 |
